# Supplementary material for: Large-scale stochastic simulation of open quantum systems
Source: Nat Commun. 2025 Dec 10;16:11074. doi: 10.1038/s41467-025-66846-x (PMC12698786; doi:10.1038/s41467-025-66846-x)
Supplement: Supplementary file 1 — Supplementary Information [file 41467_2025_66846_MOESM1_ESM.pdf]

# Supplementary information: Large-scale stochastic simulation of open quantum systems

## 1. Proof of equivalence of MCWF and Lindblad master equation

**Theorem 1** (Equivalence of MCWF and Lindblad master equation)**.** *Given the Lindblad master equation as in Eq. (1) with solution $\rho(t)$, and the MCWF Hamiltonian defined as*

$$H = H_0 - \frac{i\hbar}{2}\sum_{m=1}^{k}\gamma_m L_m^\dagger L_m, \tag{1}$$

*consider the following: Let $|\psi_i(t)\rangle$ for $i = 1, \dots, N$ be state vector trajectories sampled from the initial state $\rho(0)$. The average of the outer products of these sampled pure states at time $t$ is given by*

$$\bar{\mu}_N(t) = \frac{1}{N}\sum_{i=1}^{N} |\psi_i(t)\rangle\langle\psi_i(t)|\,. \tag{2}$$

*If the time step $\delta t$ converges to 0, it holds that*

$$\rho(t) = \lim_{N\to\infty}\bar{\mu}_N(t) \qquad \forall t. \tag{3}$$

*Proof.* For $N \to \infty$, a time-evolved state is described by combining the possibilities of a jump occurring or not such that

$$\begin{aligned}\bar{\mu}(t+\delta t) &= (1-\delta p)\frac{U(\delta t)\bar{\mu}(t)U^\dagger(\delta t)}{\sqrt{1-\delta p}\sqrt{1-\delta p}} + \sum_{m=1}^{k}\delta p_m \frac{L_m\bar{\mu}(t)L_m^\dagger}{\sqrt{\delta p_m}\sqrt{\delta p_m}/(\gamma_m\delta t)}\\ &= \bar{\mu}(t) - iH\delta t\bar{\mu}(t) + \bar{\mu}(t)iH^\dagger\delta t + \delta t\sum_{m=1}^{k}\gamma_m L_m\bar{\mu}(t)L_m^\dagger + \mathcal{O}(\delta t^2),\end{aligned} \tag{4}$$

where we have used the definition of the matrix exponential up to the second summand $U(\delta t) = e^{-i\delta t H} = 1 - iH\delta t + \mathcal{O}(\delta t^2)$. Taking the derivative such that the LHS of the master equation is created, it follows that

$$\begin{aligned}\frac{d}{dt}\bar{\mu}(t) &= \lim_{\delta t\to 0}\frac{\bar{\mu}(t+\delta t)-\bar{\mu}(t)}{\delta t}\\ &= \lim_{\delta t\to 0}\left(-iH\bar{\mu}(t) + \bar{\mu}(t)iH^\dagger \quad + \sum_{m=1}^{k}\gamma_m L_m\bar{\mu}(t)L_m^\dagger + \mathcal{O}(\delta t)\right)\\ &= -iH\bar{\mu}(t) + \bar{\mu}(t)iH^\dagger \quad + \sum_{m=1}^{k}\gamma_m L_m\bar{\mu}(t)L_m^\dagger + \lim_{\delta t\to 0}(\mathcal{O}(\delta t))\\ &= -iH\bar{\mu}(t) + \bar{\mu}(t)iH^\dagger \quad + \sum_{m=1}^{k}\gamma_m L_m\bar{\mu}(t)L_m^\dagger\\ &= -i\left(H_0 - \frac{i\hbar}{2}\sum_{m=1}^{k}\gamma_m L_m^\dagger L_m\right)\bar{\mu}(t) + \bar{\mu}(t)i\left(H_0 + \frac{i\hbar}{2}\sum_{m=1}^{k}\gamma_m L_m^\dagger L_m\right) + \sum_{m=1}^{k}\gamma_m L_m\bar{\mu}(t)L_m^\dagger\\ &= -i[H_0,\bar{\mu}(t)] - \sum_{m=1}^{k}\gamma_m\left(L_m\bar{\mu}(t)L_m^\dagger - \frac{1}{2}\left\{L_m^\dagger L_m, \bar{\mu}(t)\right\}\right).\end{aligned} \tag{5}$$

This has the form of the RHS of the master equation such that for a sufficiently small time step $\delta t$ we have that

$$\rho(t) = \lim_{N\to\infty}\frac{1}{N}\sum_{j=1}^{N}|\psi_j(t)\rangle\langle\psi_j(t)|\,, \quad \forall t. \tag{6}$$

Since for a fixed $t \in [0, T]$ every sample $|\psi_j(t)\rangle\,, j = 1, \dots, N$ is independent and identically distributed, it follows from the law of large numbers that $\rho(t) = \mathbb{E}[\overline{\mu}_N(t)]$ for all $N, t$. □

| Method | Time Evolution | Storage | Exp. Value |
|---|---|---|---|
| Lindblad | $\mathcal{O}(nd^{6L})$ | $\mathcal{O}(d^{2L})$ | $\mathcal{O}(d^{6L})$ |
| MCWF | $\mathcal{O}(Nnd^{3L})$ | $\mathcal{O}(Nd^{L})$ | $\mathcal{O}(Nd^{4L})$ |
| MPO | $\mathcal{O}(nLd^4D_H^2D_s^2)$ | $\mathcal{O}(Ld^2D_s^2)$ | $\mathcal{O}(Ld^2D_s^3)$ |
| TJM | $\mathcal{O}(NnL\chi_{\text{max}}^3[dD+d^2])$ | $\mathcal{O}(NLd\chi_{\text{max}}^2)$ | $\mathcal{O}(NLdD\chi_{\text{max}}^3)$ |

Supplementary Table I: This table compares the complexities between each method, including the time to generate the time-evolution, store the final data structure, and calculate expectation values with the result. These are dependent on the physical dimension $d$, system size $L$, time steps $n$, trajectories $N$, MPS bond dimension $\chi_{\text{max}}$, system MPO bond dimension $D$, density matrix bond dimension $D_s$, and Hamiltonian bond dimension $D_H$ according to the $W^{II}$ algorithm [1, 2]. Note that this assumes that all information is kept, despite the TJM being embarassingly parallel, where the individual trajectories could be used to calculate an expectation value and then be discarded in most practical contexts.

### 2. Frobenius variance

We present a lemma that is helpful in proving Monte Carlo convergence in the main text.

**Lemma 2** (Frobenius variance)**.** *Let $X, Y \in \mathbb{C}^{n\times n}$ be uncorrelated random matrices with $\mathbb{E}[X] = \mathbb{E}[Y] = A$ and the Frobenius norm for a squared complex matrix $A \in \mathbb{C}^{n,n}$ be given as $\|A\|_F = \sqrt{Tr(A^\dagger A)} = \sum_{i,j} |a_{i,j}|^2$. Then, the variance according to the Frobenius norm is given by*

$$\mathbb{V}_F[X] = \mathbb{E}\left[\|X - \mathbb{E}[X]\|_F^2\right], \tag{7}$$

*and it holds true that*

*i)* $\mathbb{V}_F(X+Y) = \mathbb{V}_F(X) + \mathbb{V}_F(Y)$,

*ii) for any scalar* $a \in \mathbb{R}$, $\mathbb{V}_F(aX) = a^2\mathbb{V}_F(X)$.

The proof of Lemma 2 is straightforward and is presented subsequently.

**Definition 3** (Density matrix variance and standard deviation)**.** *Let $\|\cdot\|$ be a matrix norm, and let $X \in \mathbb{C}^{n\times n}$ be a matrix-valued random variable defined on a probability space $(\Omega, \mathcal{F}, \mathbb{P})$, where $\mathbb{P}$ is a probability measure. The variance of $X$ with respect to the norm $\|\cdot\|$ is defined as*

$$\mathbb{V}[X] = \mathbb{E}\left[\|X - \mathbb{E}[X]\|^2\right], \tag{8}$$

*where $\mathbb{E}[X]$ denotes the expectation of $X$. The expectation $\mathbb{E}[X]$ is computed entrywise with each entry being the expectation according to the respective marginal distributions of the entries. Specifically, for each $i, j \in \{1, \dots, n\}$,*

$$\mathbb{E}[X]_{i,j} = \mathbb{E}_{\mathbb{P}_{i,j}}[x_{i,j}], \tag{9}$$

*where $x_{i,j}$ is the $(i,j)$-th entry of the matrix $X$, and $\mathbb{P}_{i,j}$ is the marginal distribution of $x_{i,j}$ induced by $\mathbb{P}$. The expectation value of the norm of a matrix $\mathbb{E}[\|\cdot\|]$ is defined as the multidimensional integral over the function $\|\cdot\| : \mathbb{C}^{n,n} \mapsto \mathbb{R}$ according to its marginal distributions $\mathbb{P}_{i,j}$. The standard deviation of $X$ with respect to the norm $\|\cdot\|$ is then defined as*

$$\sigma(X) = \sqrt{\mathbb{V}[X]} = \sqrt{\mathbb{E}\left[\|X - \mathbb{E}[X]\|^2\right]}. \tag{10}$$

In what follows, we make use of the Frobenius norm, defined for a squared complex matrix $A \in \mathbb{C}^{n,n}$ as $\|A\|_F := \sqrt{\text{Tr}(A^\dagger A)} = \sum_{i,j} |a_{i,j}|^2$.

**Lemma 4** (Frobenius variance)**.** *Let $X, Y \in \mathbb{C}^{n,n}$ be uncorrelated random matrices with $\mathbb{E}[X] = \mathbb{E}[Y] = A$. Then the variance according to the Frobenius norm is given as*

$$\mathbb{V}_F[X] = \mathbb{E}\left[\|X - \mathbb{E}[X]\|_F^2\right], \tag{11}$$

*and it holds true that*

*i)* $\mathbb{V}_F(X+Y) = \mathbb{V}_F(X) + \mathbb{V}_F(Y)$,

*ii) for any scalar $a \in \mathbb{R}$, $\mathbb{V}_F(aX) = a^2\mathbb{V}_F(X)$.*

*Proof.* First, we prove that $\mathbb{V}_F[X+Y] = \mathbb{V}_F[X] + \mathbb{V}_F[Y]$ when $X$ and $Y$ are uncorrelated random matrices. The variance for a random matrix $X$ with respect to the Frobenius norm is defined as

$$\mathbb{V}_F[X] = \mathbb{E}[\|X - A\|_F^2] = \mathbb{E}\left[\sum_{i,j}^{n} |x_{i,j} - a_{i,j}|^2\right], \tag{12}$$

where $A = \mathbb{E}[X]$ and $a_{i,j}$ are the elements of $A$. Similarly, the variance for $Y$ is

$$\mathbb{V}_F[Y] = \mathbb{E}[\|Y - A\|_F^2] = \mathbb{E}\left[\sum_{i,j}^{n} |y_{i,j} - a_{i,j}|^2\right]. \tag{13}$$

To find the variance of $X+Y$, note that $\mathbb{E}[X+Y] = \mathbb{E}[X] + \mathbb{E}[Y] = 2A$. Therefore,

$$\begin{aligned}\mathbb{V}_F[X+Y] &= \mathbb{E}\left[\|(X+Y) - \mathbb{E}[X+Y]\|_F^2\right] \\ &= \mathbb{E}\left[\sum_{i,j}^{n} |(x_{i,j} + y_{i,j}) - 2a_{i,j}|^2\right].\end{aligned} \tag{14}$$

Expanding the squared term, we have

$$\begin{aligned}|(x_{i,j} + y_{i,j}) - 2a_{i,j}|^2 &= |\left((x_{i,j} - a_{i,j}) + (y_{i,j} - a_{i,j})\right)|^2 \\ &= |x_{i,j} - a_{i,j}|^2 + |y_{i,j} - a_{i,j}|^2 \\ &\quad + 2\,\mathrm{Re}\left((x_{i,j} - a_{i,j})\overline{(y_{i,j} - a_{i,j})}\right).\end{aligned}$$

Taking the expectation and using the fact that $X$ and $Y$ are uncorrelated, we get

$$\begin{aligned}\mathbb{E}\left[|(x_{i,j} - a_{i,j}) + (y_{i,j} - a_{i,j})|^2\right] &= \mathbb{E}\left[|x_{i,j} - a_{i,j}|^2\right] + \mathbb{E}\left[|y_{i,j} - a_{i,j}|^2\right] \\ &\quad + 2\mathbb{E}\left[\mathrm{Re}((x_{i,j} - a_{i,j})\overline{(y_{i,j} - a_{i,j})})\right].\end{aligned} \tag{15}$$

As with $X$ and $Y$, $x_{i,j}$ and $y_{i,j}$ are also uncorrelated for all $i, j = 1, \ldots, n$ and hence $\mathbb{E}\left[\mathrm{Re}((x_{i,j} - a_{i,j})(y_{i,j} - a_{i,j}))\right] = 0$. Thus, this reduces to

$$\mathbb{E}\left[(x_{i,j} - a_{i,j})^2\right] + \mathbb{E}\left[(y_{i,j} - a_{i,j})^2\right]. \tag{16}$$

Summing over all elements $(i, j)$, we get

$$\begin{aligned}\mathbb{V}_F[X+Y] &= \sum_{i,j}^{n} \mathbb{E}\left[(x_{i,j} - a_{i,j})^2\right] + \sum_{i,j}^{n} \mathbb{E}\left[(y_{i,j} - a_{i,j})^2\right] \\ &= \mathbb{V}_F[X] + \mathbb{V}_F[Y].\end{aligned} \tag{17}$$

Next, we prove that $\mathbb{V}_F[aX] = a^2\mathbb{V}_F[X]$ for any scalar $a \in \mathbb{R}$. The variance for $aX$ is

$$\mathbb{V}_F[aX] = \mathbb{E}[\|aX - \mathbb{E}[aX]\|_F^2]. \tag{18}$$

Since $\mathbb{E}[aX] = a\mathbb{E}[X] = aA$, we have

$$\mathbb{V}_F[aX] = \mathbb{E}\left[\|aX - aA\|_F^2\right]. \tag{19}$$

Factoring out $a$ from the Frobenius norm, we get

$$\|aX - aA\|_F = |a|\|X - A\|_F, \tag{20}$$

and thus

$$\|aX - aA\|_F^2 = a^2\|X - A\|_F^2. \tag{21}$$

Taking the expectation, we obtain

$$\mathbb{V}_F[aX] = \mathbb{E}[a^2\|X - A\|_F^2] = a^2\mathbb{E}[\|X - A\|_F^2] = a^2\mathbb{V}_F[X]. \tag{22}$$

Therefore, we have shown that for any scalar $a \in \mathbb{R}$,

$$\mathbb{V}_F[aX] = a^2\mathbb{V}_F[X]. \tag{23}$$

□

**Theorem 5** (Convergence of TJM). *Let $d \in \mathbb{N}$ be the physical dimension and $L \in \mathbb{N}$ be the number of sites in the open quantum system described by the Lindblad master equation. Furthermore, let $\boldsymbol{\rho}_N(t) = \frac{1}{N}\sum_{j=1}^N |\boldsymbol{\Psi_j}(t)\rangle\langle\boldsymbol{\Psi_j}(t)|$ be the positive semi-definite approximation of the solution $\rho(t)$ of the Lindblad master equation in MPO format at time $t \in [0,T]$ for some ending time $T > 0$ and $N \in \mathbb{N}$ trajectories, where $|\boldsymbol{\Psi_j}(t)\rangle$ is a trajectory sampled according to the TJM in MPS format of full bond dimension. Then, the expectation value of the approximation of the corresponding density matrix $\rho_N(t) \in \mathbb{C}^{d^L,d^L}$ is given by $\rho(t)$ and there exists a $c > 0$ such that the standard deviation of $\rho_N(t)$ can be upper bounded by*

$$\sigma(\rho_N(t)) \leq \frac{c}{\sqrt{N}} \tag{24}$$

*for all matrix norms $\|\cdot\|$ defined on $\mathbb{C}^{d^L,d^L}$.*

*Proof.* For a sufficiently small time step $\delta t$, it can be shown that the average of the trajectories converges to the solution $\rho(t)$ of the Lindblad equation as the number $N$ of trajectories approaches infinity, for all $t$, namely

$$\lim_{N\to\infty}\frac{1}{N}\sum_{j=1}^N |\psi_j(t)\rangle\langle\psi_j(t)| = \lim_{N\to\infty}\rho_N(t) = \rho(t), \quad t \in [0,T]. \tag{25}$$

From Theorem 1 we know that $\rho(t) = \mathbb{E}[\rho_N(t)]$ for all $N, t$. Additionally, let $X(t) = X_1(t)$. We know that $\mathbb{V}_F[X(t)]$ is bounded since every realization of $X(t)$ and $\mathbb{E}[X(t)]$ are density matrices, so they have trace 1. Thus, we have that $0 \leq \|\rho_1 - \rho_2\|_F \leq 2$ for all density matrices $\rho_1, \rho_2 \in \mathcal{B}(\mathcal{H})$ regardless of the system size. Each of the $N$ summands has the core structure of Eq. (3) in Ref. [3] with $d_k = 1$, which guarantees the positive semi-definiteness of each term and hence of the full operator.

To get a realization $X(t)$ for a certain $t \in [0,T]$, we simulate a trajectory from $X(0)$ by choosing a sufficiently small discretization $\delta t$ and stop the simulation at $X(t)$. Now it is easy to check that the variance of $\mathbb{V}_F[\rho_N(t)]$ decreases linearly with $N$. Concretely,

$$\begin{aligned}\mathbb{V}_F[\rho_N(t)] &= \mathbb{V}_F\left[\frac{1}{N}\sum_{i=1}^N X_i(t)\right] = \frac{1}{N^2}\mathbb{V}_F\left[\sum_{i=1}^N X_i(t)\right] \\ &= \frac{1}{N^2}\sum_{i=1}^N \mathbb{V}_F\left[X_i(t)\right] = \frac{1}{N}\mathbb{V}_F[X(t)] \qquad (26) \\ &\leq \frac{4}{N}, \qquad (27)\end{aligned}$$

where we have used that $\mathbb{V}_F[aX(t)] = a^2\mathbb{V}_F[X(t)]$, $\mathbb{V}_F[X_1(t)+X_2(t)] = \mathbb{V}_F[X_1(t)] + \mathbb{V}_F[X_2(t)]$ and the fact that the $X_i(t)$ are independent samples and identically distributed. Thus, the Frobenius norm standard deviation is upper bounded by

$$\sigma_F[\rho_N(t)] = \frac{1}{\sqrt{N}}\sigma_F[X(t)] \leq \frac{2}{\sqrt{N}}. \tag{28}$$

By the equivalence of norms on finite vector spaces, there exists $c_1, c_2 \in \mathbb{R}$ such that $c_1\|A\|_F \leq \|A\| \leq c_2\|A\|_F$ for all complex square matrices $A$ and all matrix norms $\|\cdot\|$. Thus, the convergence rate $\mathcal{O}(1/\sqrt{N})$ also holds true in trace norm and any other relevant matrix norm and is independent of system size. The proposition follows directly. □

### 3. Complexity of TJM

For a TJM procedure we sample $N \in \mathbb{N}$ trajectories, each with $n = \frac{T}{\delta t} \in \mathbb{N}$ time steps, where $T \in \mathbb{R}_+$ is the terminal time and $\delta t$ the time step size. Since each time step consists of the calculation of the probability over the jump operators, jump application, TDVP step, and a dissipative contraction, the total complexity can be calculated as

$$\mathcal{O}(Nn(\text{probability distribution} + \text{ jump application} + \text{TDVP} + \text{Dissipative contraction})). \tag{29}$$

For the complexity calculation, we consider $\chi_{\text{max}}$ as the maximum bond dimension of the MPS $|\boldsymbol{\Psi(t)}\rangle$, $d$ the dimension of the local Hilbert space, $D$ as the maximum bond dimension of the MPO representing the Hamiltonian $H_0$ of the closed system.

The calculation of the probability distribution can be seen as an efficient sweep across the MPS $|\boldsymbol{\Psi(t)}\rangle$, where at each site $\ell = 1, \dots, L$ we have to contract the jump operators $L_j^{[\ell]}, j \in S(\ell)$ into the $\ell$-th site tensor and calculate the inner product of the MPS which requires $\mathcal{O}(k\chi_{\text{max}}^3 d)$ operations since it has to be done for every jump operator $L_m, m = 1, \dots, k$. The sampling of an $\epsilon \in [0, 1]$, which is uniformly distributed has complexity $\mathcal{O}(1)$. It is like sampling a jump operator according to the distribution $\Pi(t)$ [4]. Since $L_m$ are single-site operators, the jump application is just a contraction of a matrix in $\mathbb{C}^{d\times d}$ into a site tensor of $|\boldsymbol{\Psi(t)}\rangle$, which takes $\mathcal{O}(\chi_{\text{max}}^2 d^2)$ operations.

The complexity of the 2TDVP is given by $\mathcal{O}(L(\chi_{\text{max}}^2 d^3 D^2 + \chi_{\text{max}}^3 d^2 D + \chi_{\text{max}}^3 d^3))$, whereas the single-site version scales with $\mathcal{O}(L(\chi_{\text{max}}^2 d^2 D^2 + \chi_{\text{max}}^3 dD + \chi_{\text{max}}^3 d^2))$. Since in TJM the 2TDVP is not performed with maximum bond dimension $\chi_{\text{max}}$, the 1TDVP complexity is of primary interest. In the dissipative contraction $\mathcal{D}[\delta t]$, each of the $L$ site tensors has to be contracted with a single site tensor $D_\ell \in \mathbb{C}^{d,d}$, which requires $\mathcal{O}(d^2\chi_{\text{max}}^2)$ operations, leading to a complexity of $\mathcal{O}(Ld^2\chi_{\text{max}}^2)$ for the dissipative contraction.

Collecting the above considerations, the total complexity of the TJM is given as

$$\begin{aligned}\mathcal{O}\bigg(&Nn\big[L(d^2D^2\chi_{\text{max}}^2 + dD\chi_{\text{max}}^3 + d^2\chi_{\text{max}}^3 \\ &+ d^2\chi_{\text{max}}^2) + kd\chi_{\text{max}}^3 + d^2\chi_{\text{max}}^2\big]\bigg).\end{aligned} \tag{30}$$

Dominant terms are the squared physical dimension $d$, the cubic bond dimension $\chi_{\text{max}}$ of the MPS in the TDVP sweep and the product $dD$ of the physical dimension and the bond dimension of the MPO, such that the shorthand complexity of TJM is

$$\mathcal{O}\bigg(NnL\chi_{\text{max}}^3\big[dD + d^2\big]\bigg). \tag{31}$$

[1] M. P. Zaletel, R. S. K. Mong, C. Karrasch, J. E. Moore, and F. Pollmann, Time-evolving a matrix product state with long-ranged interactions, Phys. Rev. B **91**, 165112 (2015).

[2] H. Landa and G. Misguich, Nonlocal correlations in noisy multiqubit systems simulated using matrix product operators, SciPost Phys. Core **6**, 037 (2023).

[3] F. Verstraete, J. J. García-Ripoll, and J. I. Cirac, Matrix product density operators: Simulation of finite-temperature and dissipative systems, Phys. Rev. Lett. **93**, 207204 (2004).

[4] A. J. Walker, An efficient method for generating discrete random variables with general distributions, ACM Trans. Math. Soft. **3**, 253 (1977).
